# Peer-based behavioral health program for drug users in China: a pilot study

**DOI:** 10.1186/1471-2458-11-693

**Published:** 2011-09-07

**Authors:** Song-Ying Shen, Zhou-Bin Zhang, Joseph D Tucker, Helena Chang, Guan-Rong Zhang, Ai-Hua Lin

**Affiliations:** 1 Department of Medical Statistics and Epidemiology, School of Public Health, Sun-Yat-sen University, Guangzhou, PR China; 2 Guangzhou Center for Disease Control and Prevention, Guangzhou, PR China; 3 Infectious Disease Unit, Massachusetts General Hospital, Boston, MA, USA; 4 School of Medicine and Public Health, University of Wisconsin, Madison, WI, USA

## Abstract

**Background:**

Many injection drug users (IDUs) in China have high risk sexual behaviors that contribute to the spread of HIV infection. Although many IDUs in China move through drug rehabilitation centers, this opportunity for sexual health education has largely been overlooked.

**Methods:**

A convenience sample of 667 drug users from two rehabilitation centers in South China was recruited in the study. Two hundred and forty seven drug users from a single Guangdong Province rehabilitation center received the peer-based education intervention, while 420 drug users from another rehabilitation center received routine HIV/STI education and was used as the control. One hundred and eighty nine (22.1%) individuals refused to participate in the study. HIV/STI behavioral and knowledge domains were assessed at 3 months in rehabilitation centers after the intervention (first follow-up) and at 2-23 months in the community after release (second follow-up).

**Results:**

Drug users who completed the intervention reported more frequent condom use with casual sex partners (60.0% vs. 12.5% condom use every time, p = 0.011) and less frequent injection (56.7% vs. 26.4% no injection per day, p = 0.008) at the second follow-up compared to those in the routine education group. Loss to follow up was substantial in both control and intervention groups, and was associated with living far from the detention center and having poor HIV knowledge at baseline.

**Conclusions:**

This study shows that rehabilitation centers may be a useful location for providing behavioral HIV/STI prevention services and referral of individuals to community-based programs upon release. More research is needed on behalf of detained drug users in China who have complex social, medical, and legal needs.

## Background

China's HIV epidemic has shifted from that of predominately injection drug users' (IDUs) to one that is predominately sexually transmitted [1-6]. Many individuals in China have both unsafe sex and unsafe drug use [7-9], making sexual health services for drug users an important area for HIV/STI prevention services. In South China, the sexually transmitted HIV epidemic and drug use HIV epidemic have substantial overlap. IDUs in China have been shown to have a higher prevalence of sexually transmitted infections compared to non-drug using individuals [10,11] while patients at STI clinics have a higher prevalence of drug use compared to other individuals [12]. A population-based study of HIV infection at southern Chinese STI clinics found that drug use was the strongest predictor of HIV infection (odds ratio = 25) [12], confirming the importance of understanding sexual health among IDUs.

China's dramatic response to the IDU HIV epidemic provides unique opportunities for both community-based and detention-based HIV prevention programs. By the end of 2008, more than 1000 methadone maintenance centers and syringe exchange programs had been established in China [13]. Drug users can enter drug rehabilitation centers either voluntarily or following arrest in China [14]. However, drug users' lack of trust in public health authorities at rehabilitation centers complicates behavioral interventions in this context [15].

Peer-based programs may be especially useful in this context since they may build trust between service providers and drug users. Building on previously formed social networks and healthy network norms can improve the feasibility and sustainability of these approaches. Peer-based interventions use these social relationships to guide proscriptive behavioral norms promoting safe sex [16]. Peer-based HIV prevention programs have been effective within China [17-20] and outside of China [21-23]. In China, studies of peer education interventions (PEI) for young people indicate that peer education can increase HIV-related knowledge, attitudes and behavior intentions after intervention [24-26]. There are also some studies that attempt to evaluate peer education programs for IDUs in the community in China. Many Chinese cities include peer education interventions as a part of their needle and syringe exchange programs (NEP) [17-20]. Most of the studies have found that HIV-related knowledge increased after participation in these programs. However these studies were limited by lack of a control group [17] or no follow-up [18].

This study used the infrastructure of a drug user rehabilitation center in South China to offer a peer-based HIV/AIDS behavioral intervention for drug users. This pilot study compared a peer-led intervention at a single rehabilitation center to routine HIV/STI education at another nearby rehabilitation center, with follow up of IDUs both while in detention and in the community post-incarceration.

## Methods

### Subjects

Guangdong Province in southern China was chosen for this study because of recent increases in reported sexually transmitted HIV infection [27] and other STIs [28]. This southern Chinese province has a total population of 95 million and a disproportionate share of rural to urban migrants [29]. The capital city of Guangdong Province, Guangzhou City, was chosen to conduct this study because of existing connections to HIV prevention programs and willingness on the part of the detention centers to participate. A convenience sample of two compulsory drug rehabilitation centers in Guangzhou City was selected from the nine total drug rehabilitation centers in the region in July 2006. Although there is substantial variation in the implementation of drug law in China, typically those detained for illegal drug use undergo a six month compulsory substance abuse treatment in a drug rehabilitation center. These detention centers, administratively separate from the legal system and the public health system, are run by local police [30]. All individuals who enter the drug rehabilitation system are provided HIV and STI testing and counseling [31,32].

Study subjects were enrolled by public health researchers separately from the detention staff personnel with the clear understanding that their participation in this research project would not affect their future ability to access HIV/STI services. Study eligibility requirements included the following: entrance to one of the two selected drug rehabilitation centers between May and July of 2006, first-time offenders, and not having participated in other peer-based HIV education programs. Repeat offenders were excluded because they receive more extensive education and counseling services on a routine basis. The research project was explained to each of the participants and verbal informed consent was obtained. Each informed consent had at least one witness who was the staff of the centre to confirm that this study was not compulsory. There were no inducements to participate. All research procedures were reviewed and approved by the Sun Yat-Sen University Ethical Review Board.

### Study design

**Figure 1:**
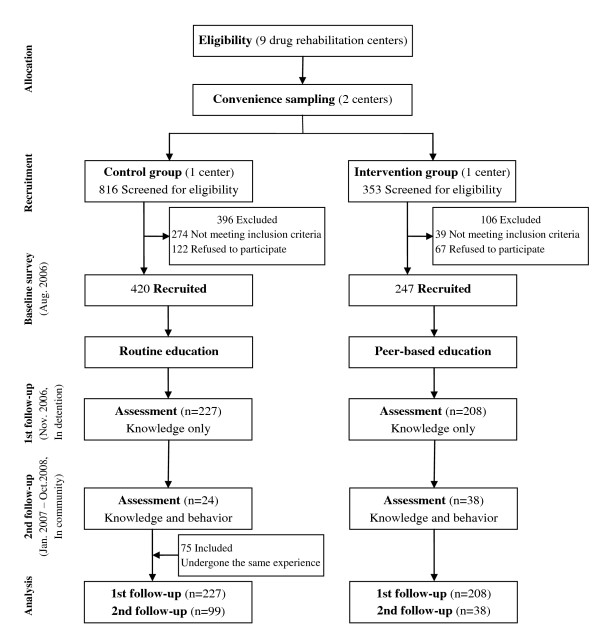
**Study design and subject flow chart**.

This pilot intervention evaluated peer-based HIV/STI behavioral interventions at two drug rehabilitation centers. Eligible drug users at the drug rehabilitation center from one district were selected as the intervention group and those detained in a neighboring district were the control group. Figure 1 shows the study design. The total number of individuals who refused or were excluded was recorded for each of the two sites. In the control research site, a total of 816 individuals entered the system between May and July of 2006, 274 of whom were repeat offenders and thus not eligible. Among all eligible participants, 420 individuals agreed to participate and an estimated 122 (22.5%) of individuals refused to participate. At the intervention site, there were 353 individuals entered during the period of recruitment. Among them, 39 drug users were ineligible because they were repeat offenders. A total of 247 individuals participated at the intervention site at baseline, and an estimated 67 individuals (21.3%) refused to participate. Exact numbers of those who refused to participate are not available because ineligibility and refusal were not separated at the time of study accrual. At the control site, routine HIV/STI education consisted of police personnel handing out educational pamphlets and providing lectures focused on HIV/STI education. At the intervention site, a peer education program for HIV/AIDS prevention was implemented (a more detailed description follows). The first follow-up survey was conducted approximately three months after the baseline survey while individuals were still in the rehabilitation centers, and included a test of HIV/STI knowledge (Intervention group (n = 208) vs. Control group (n = 227)). The second follow-up survey was done when participants were in the community after release from rehabilitation centers, ranging from 2 to 23 months after the first follow up (Intervention group (n = 38) vs. Control group (n = 24)). The loss to follow up rate was higher than expected. To ensure sufficient statistical power, an additional 75 drug users not included in the original control group were added in the control group at the second follow-up. The purpose was to make up the large percentage of lost follow-up. These 75 individuals were recruited in the same center of control group at the time of second follow-up. All of them were being detained for the second time. The rationale for including these individuals includes the following: (1) they received routine HIV/STI education counselling similar to the control group, (2) they were also released and in the community for a period of time comparable to drug users in the control group, (3) if we chose repeat detained drug users, they would receive traditional education more than once, (4) the behaviours of the 75 subjects when they were in the community were not significantly different with the 24 persons in the original control group at the second follow-up [33]. Using a structured questionnaire, face-to-face interviews with trained research personnel were conducted at each survey. The interview items were developed by the study team and were pre-tested among detained drug users from the same city. More detailed information regarding the interview instrument has been published previously [34].

### Intervention methods

Intervention education materials were based on guidelines issued by the Chinese Ministry of Health that focus on harm reduction among drug users [35]. The intervention was based on a social learning theory conceptual framework. Social learning theory holds that individuals must have the opportunity to observe and practice modeled behavior before they can feel confident to perform it effectively. The use of credible role models, such as fellow drug users, who have experienced similar circumstances and have developed the skills to make lifestyle changes, can be far more effective in bringing about and reinforcing behavior change than ongoing contact with a professional counselor [36]. At the intervention site, the peer-based behavioral intervention was organized by one physician, one nurse, and one coordinator. The physician, nurse, and coordinator supervised, advised and provided counselling to the drug users/peer groups. The intervention program consisted of three stages: recruitment, training peer educators, and implementation of HIV prevention activities.

During recruitment, the physician selected one to two drug users who appeared to be respected by their inmates to voluntarily lead peer behavioral intervention groups. A total of 34 drug users were selected as the peer leader candidates. Twenty-nine drug users agreed and were trained as peer educators while the other five individuals declined to participate. The intervention focused on teaching peer educators HIV knowledge and risk reduction information as well as how to educate fellow inmates. The two two-hour training sessions included information in two domains: (1) the history of HIV, HIV in China and Guangzhou, HIV transmission routes, prevention measures and the vital role peer educators play in preventing further disease spread; (2) communication skills, self-esteem, decision-making skills, assertiveness, safer injection and sexual practices and planning prevention activities. The training sessions were led by a physician from the Guangzhou Center for Disease Control and Prevention. To complete the training course, the peer educators had to a take a final graded qualification exam. After the training, peer educators received information pamphlets for self-study and review. Finally, peer educators conducted group education activities cell by cell. The intervention was implemented in five meeting sessions from September 2006 to November 2006, with a total educating duration of ten hours. Peer educators were supervised and reviewed by either the project physician or the nurse. The principal content included: (1) making educational posters to display in cells; (2) delivering one-on-one training to peers; (3) engaging in exercises focusing on injection and sexual risk behaviors; (4) role-playing and related activities; and (5) activities designed to get participants to practice new skills (two hours per meeting for group work).

### Sample size

The sample size was calculated by use of the usual formula for continuous outcome *n *= 4(*t_α/2 _*+ *t_β_*)^2 ^*S*^2^/*δ^2^*, where *S *was the sample standard deviation of HIV knowledge score among two groups. Assuming Type I error *α *= 0.05 and the power level 1-*β *= 0.9, 205 subjects were required for each group to detect a difference (*δ*) of 1.19 in HIV knowledge score between the intervention and control group at post-intervention test in a former study [37], based on *S *= 3.62 and an anticipated lost to follow up rate of 5%. However, the loss to follow up rate was higher than expected, and so an additional 75 drug users not included in the original control group were added to the control group.

### Outcome measures

Self-reported measures of knowledge and behaviors were obtained at the time of baseline and two follow-up surveys. At baseline, demographic information, HIV knowledge, and HIV risk behavioral data were collected. The first follow-up, conducted while in detention, reassessed HIV knowledge. The second follow-up, conducted in the community, focused on injection and sexual risk behaviors.

Three domains in HIV knowledge were assessed: (1) basic HIV knowledge (11 items), (2) HIV risk reduction (14 items) and (3) condom usage (9 items). Each correct answer was scored one point. With a total of 34 questions, a full score with all the questions answered correctly was 34.

### Statistical analyses

The Statistical Package for the Social Sciences (SPSS Statistics 17.0.1; SPSS Inc., Chicago, IL) and the Statistical Analysis System (SAS 9.1.3; SAS Institute Inc., Cary, NC) were used for data analysis. Descriptive statistics, such as frequency, were applied for categorical variables; mean and standard deviation were used for continuous variables. Binary variables and multivariate items were compared by *chi-*squared tests. Ordinal categorical variables were compared using nonparametric tests. Continuous variables were compared by *t*-test or nonparametric tests, depending on the distribution of the data. Paired *t*-tests were used to compare the mean score differences of responses to HIV questions between baseline and each follow-up assessment in the same group. In order to assess intervention effects, we compared knowledge score changes over time. Although groups were found to be comparable on key outcome measures at baseline, we were concerned that several variables were different between the intervention and control group. Therefore, we adjusted analysis for baseline knowledge and potential covariates that had been found different for the two groups. Linear mixed regression model with random individual effects was employed, with individuals at level 2 and repeated measurements at level 1, to obtain estimates of mean score changes in HIV-related knowledge for the intervention and control group, as well as corresponding between groups differences of changes. This approach allows the inclusion of individuals with missing measures, thus minimizing bias. Consistent with the intention-to-treat principle, we performed analysis including all available cases, with neither conducting a complete cases analysis nor imputing data. An alpha of 0.05 to assess for significance was used for all statistical tests.

## Results

### Comparability of characteristics between intervention and control groups at baseline

Two hundred and eight participants in the intervention group and 227 in the control group were included in the baseline analysis. The mean age of participants was 29.5 years old (+/-6.7) in the intervention group and 33.7 years old (+/-8.3) in the control group. The two groups did not differ in regards to marital status, occupation, home province, or monthly income (Table 1). There were significantly more females in the control group compared to the intervention group (34.4% vs. 11.5%, p < 0.001). Participants in the intervention group had significantly less education compared to the control group (p = 0.002). Drug users in the control group were significantly more likely to be Han ethnicity compared to the intervention group (p = 0.043). At the second follow-up, there were 38 people in the intervention group and 24 in the control group. An additional 75 drug users were added in the control group to make up its samples of 99 at the second follow-up.

**Table 1:** Baseline socio-demographic characteristics of participants in the two groups

	Intervention group *n*(%) (*n *= 208)	Control group *n*(%) (*n *= 227)	Total *n*(%) (*n *= 435)	*χ*^2^/*Z*	p-value
**Sex**					
Male	184(88.5)	149(65.6)	333(76.6)	31.497	< 0.001
Female	24(11.5)	78(34.4)	102(23.4)		
**Marital status**					
Unmarried	74(35.6)	71(31.4)	145(33.4)		
Married	77(37.0)	72(31.9)	149(34.3)	4.319	0.115
Other	57(27.4)	83 (36.7)	140 (32.3)		
**Ethnicity**					
Han	190(91.3)	218(96.0)	408(93.8)	4.100	0.043
Other	18(8.7)	9(4.0)	27(6.2)		
**Education**					
Illiteracy or primary	91(43.8)	67(29.5)	158(36.3)		
Junior high school	90(43.3)	118(52.0)	208(47.8)	-3.049	0.002
High school or above	27(13.0)	42(18.5)	69(15.9)		
**Occupation**					
Laborer	48(23.1)	36(15.9)	84(19.3)		
Farmer	9(4.3)	10(4.4)	19(4.4)		
Driver	24(11.5)	17(7.5)	41(9.4)		
Businessman	32 (15.4)	42(18.5)	74(17.0)	9.973	0.126
Service	22(10.6)	38(16.7)	60(13.8)		
Unemployed	62(29.8)	65(28.6)	127(29.2)		
Other	11(5.3)	19(8.4)	30(6.9)		
**Home province**					
Guangzhou City	63(30.3)	84(37.0)	147(33.8)		
Guangdong Province (except Guangzhou)	49(23.6)	42(18.5)	91(20.9)	2.841	0.242
Other province	96(46.2)	101(44.5)	197(45.3)		
**Monthly Income (US$)**					
< 126.4	26(12.6)	23(10.2)	49(11.3)		
126.4~	57(27.5)	49(21.7)	106(24.5)	-1.451	0.147
252.9~	79(38.2)	100(44.2)	179(41.3)		
≥632.2	45(21.7)	54(23.9)	99(22.9)		

### HIV-related knowledge and behaviors at baseline

Baseline mean scores for each of the three domains for basic HIV information, HIV risk reduction, condom use and overall knowledge were 8.7, 10.0, 4.0 and 22.7 in the intervention group and 8.7, 9.8, 3.9 and 22.4 in the control group, respectively (Table 2). No significant difference existed in HIV knowledge domains between these two groups (p > 0.05). Overall, the baseline self-reported HIV risk behaviors were comparable between intervention and control groups. Over a half of participants reported injection drug use (51.0% in the intervention and 59.3% in the control). Both of the intervention and control groups had a high proportion of sharing injection equipment and unsafe sex (Table 3).

**Table 2:** Baseline and first follow-up HIV-related knowledge in the two groups

Domain	Intervention group ($\bar{X}\pm S$) (*n *= 208)			p-value^#^	Control group ($\bar{X}\pm S$) (*n *= 227)			p-value^#^
				
	Baseline	1st follow-up	Difference		Baseline	1st follow-up	Difference	
**Basic HIV information**	8.7 ± 2.2	10.6 ± 0.9	1.9 ± 2.1	< 0.001	8.7 ± 2.2	9.1 ± 2.0*	0.4 ± 1.9*	< 0.001
**HIV risk reduction**	10.0 ± 3.0	12.9 ± 2. 0	2.9 ± 3.0	< 0.001	9.8 ± 2.8	10. 0 ± 2.9*	0.2 ± 2.8*	0.322
**Condom use**	4.0 ± 2.0	6.3 ± 2.2	2.3 ± 2.5	< 0.001	3.9 ± 1.9	3.7 ± 2.0*	-0.2 ± 1.8*	0.146

**Sum**	22.7 ± 5.4	29.7 ± 3.9	7.0 ± 5.1	< 0.001	22.4 ± 5.2	22.8 ± 5.3*	0.5 ± 4.3*	0.114

*p < 0.001 for Comparison between two groups within the same assessment time point.

^# ^p-value for baseline and 1^st ^follow-up difference comparison in each group.

**Table 3:** Self-reported behaviors comparing baseline and second follow-up in the two groups

Behaviors	Baseline		*χ*^2^/*Z*	p-value	Second follow-up		*χ*^2^/*Z*	p-value
						
	Intervention group *n*(%) (*n *= 208)	Control group *n*(%) (*n *= 227)			Intervention group *n*(%) (*n *= 38)	Control group *n*(%) (*n *= 99)		
**Injection drug use**								
Yes	106(51.0)	134(59.3)	3.041	0.081	30(78.9)	72(72.7)	0.559	0.455
No	102(49.0)	92(40.7)			8(21.1)	27(27.3)		
**Frequency of injection drug use per day**								
0	6(5.7)	15(11.2)			17(56.7)	19(26.4)		
≤1	15(14.1)	26(19.4)			2(6.7)	6(8.3)		
2-4	70(66.0)	80(59.7)	-1.461	0.144	9(30.0)	41(56.9)	-2.648	0.008
> 4	12(11.3)	13(9.7)			2(6.7)	6(8.3)		
Don't know	3(2.9)	0(0.0)			0(0.0)	0(0.0)		
**Average frequency of using a new syringe**								
1	69(65.1)	82(61.2)			12(92.3)	36(67.9)		
2	22(20.7)	31(23.1)	-0.323	0.747	1(7.7)	12(22.6)	-1.789	0.074
≥3	9(8.5)	5(3.7)			0(0.0)	5(9.5)		
Refuse to answer	6(5.7)	16(12.0)			0(0.0)	0(0.0)		
**Syringe sharing**								
Yes	15(14.2)	18(13.4)			1(7.7)	3(5.7)		
No	85(80.2)	101(75.4)	0.001	0.979	12(92.3)	50(94.3)	0.076	0.783
Refuse to answer	6(5.7)	15(11.2)			0(0.0)	0(0.0)		
**Frequency of condom use at sex with steady partner**								
Never	56(47.9)	61(57.0)			12(42.9)	35(64.8)		
Sometimes	33(28.2)	18(16.8)	-0.821	0.412	14(50.0)	14(25.9)	-1.614	0.106
Every time	28(23.9)	28(26.2)			2(7.1)	5(9.3)		
**Frequency of condom use at sex with sex worker**								
Never	2(20.0)	3(16.7)			1(25.0)	2(16.7)		
Sometimes	4(40.0)	3(16.7)	-1.131	0.332	2(50.0)	6(50.0)	-	0.770
Every time	4(40.0)	12(66.7)			1(25.0)	4(33.3)		
**Frequency of condom use at sex with casual partner**								
Never	13(43.4)	12(38.7)			0(0.0)	11(68.8)		
Sometimes	4(13.3)	9(29.0)	-0.103	0.918	2(40.0)	3(18.8)	-	0.011
Every time	12(40.0)	10(32.3)			3(60.0)	2(12.5)		
Refuse to answer	1(3.3)	0(0.0)			0(0.0)	0(0.0)		

### Change in HIV-related knowledge scores during follow-ups

The intervention group had greater incremental improvement of HIV-related knowledge compared to the control group across all three domains: basic HIV information (1.9 ± 2.1 vs. 0.4 ± 1.9, p < 0.001), HIV risk reduction (2.9 ± 3.0 vs. 0.2 ± 2.8, p < 0.001), and condom use (2.3 ± 2.5 vs. -0.2 ± 1.8, p < 0.001) at the first follow-up. The intervention group scored significantly better on the first follow-up assessment than their initial baseline in all three HIV-related knowledge domains: basic HIV information (10.6 ± 0.9 vs. 8.7 ± 2.2, p < 0.001), HIV risk reduction (12.9 ± 2.0 vs. 10.0 ± 3.0, p < 0.001), and condom use (6.3 ± 2.2 vs. 4.0 ± 2.0, p < 0.001) (Table 2). But the control group showed no difference in HIV risk reduction or condom use comparing the baseline and first follow-up assessments (p > 0.05). However, compared with baseline, control drug users did score better on basic HIV information items at first follow-up survey (9.1 ± 2.0 vs. 8.7 ± 2.2, p = 0.001). At the second follow-up, the mean scores of three corresponding domains were 10.7 ± 0.6, 12.6 ± 1.7, 5.8 ± 2.2 in intervention group and 9.5 ± 1.9, 11.3 ± 2.4, 3.6 ± 2.1 in control group among the follow-up participants, respectively.

Results from the multi-level linear regression (Table 4) showed HIV-related knowledge was significantly improved for the intervention drug users at both follow-up assessments. The adjusted mean score changes from baseline to first follow-up were 7.03 for overall HIV knowledge (p < 0.001), 1.90 for basic HIV information (p < 0.001), 2.86 for HIV risk reduction (p < 0.001) and 2.27 for condom use (p < 0.001). At the second follow-up, the corresponding mean changes of each domain were 5.87, 1.67, 2.34 and 1.94 (p < 0.001), respectively. By contrast, the control group only presented minor improvements in basic HIV information at first (0.43, p = 0.001) and second (0.55, p = 0.005) follow-ups, as well as HIV risk reduction at second follow-up (0.91, p = 0.035). The increases in mean knowledge scores from baseline to both two follow-ups were greater in the intervention than in the control group (Table 4), even after adjustment for baseline differences. In addition, the intervention-control differences in mean score changes at the second follow-up were smaller than those at the first follow-up. Further analysis showed it was significant in the domain of HIV risk reduction (1.43 vs. 2.69, p = 0.015) and overall knowledge (4.67 vs. 6.60, p = 0.027).

**Table 4:** Two-level linear mixed regression on changes in mean (95% CI) scores of HIV-related knowledge in the intervention and control groups at both follow-ups

	1st follow-up in rehabilitation centers (Intervention group *n *= 208, Control group *n *= 227)				2nd follow-up in the community after release (Intervention group *n *= 38, Control group *n *= 24)			
	
	Mean changes in intervention group*	Mean changes in control group*	Between groups differences*	p-value	Mean changes in intervention group*	Mean changes in control group*	Between groups differences*	p-value
Basic HIV information	1.90 (1.63~2.17)	0.43 (0.17~0.68)	1.47 (1.10~1.85)	< 0.001	1.67 (1.35~1.99)	0.55 (0.17~0.93)	1.12 (0.62~1.62)	< 0.001
HIV risk reduction	2.86 (2.47~3.25)	0.17 (-0.21~0.54)	2.69 (2.15~3.24)	< 0.001	2.34 (1.65~3.03)	0.91 (0.06~1.75)	1.43 (0.34~2.52)	0.011
Condom use	2.27 (1.97~2.56)	-0.17 (-0.45~0.12)	2.44 (2.03~2.84)	< 0.001	1.94 (1.23~2.66)	-0.19 (-1.08~0.70)	2.13 (0.99~3.28)	< 0.001
Sum	7.03 (6.39~7.66)	0.43 (-0.18~1.04)	6.60 (5.72~7.48)	< 0.001	5.87 (4.74~7.00)	1.20 (-0.20~2.59)	4.67 (2.88~6.47)	< 0.001

*Adjusted for the baseline knowledge and potential covariates (sex, ethnicity, education status) prior the intervention.

### Self-reported HIV risk behavior comparison between the intervention and control group at the second follow-up

At the time of second follow-up, 56.7% of the intervention group versus 26.4% of the control group reported no injections per day (p = 0.008). Moreover, there was an increased reported frequency of condom use during sex with casual partners for the intervention group compared to the control group (60.0% vs. 12.5% condom use every time, p = 0.011). A difference of borderline significance (p = 0.074) was also noted with regard to the average frequency of using a new syringe among IDUs, with intervention IDUs reporting more frequently using a new syringe compared to the control group. After the intervention, the two groups had no difference in the proportion of injection drug users, syringe sharing, and condom use with steady partners or sex workers (Table 3).

### Characteristics of individuals lost to follow-up in the intervention group at second follow-up

There were no differences (p > 0.05) in terms of age, ethnicity, education, occupation, and income between retained and lost to follow-up intervention participants. Individuals who completed the second follow-up were more likely to be male (p = 0.029) and from Guangzhou (p < 0.001). Participants not lost to follow-up had significantly better basic HIV (9.3 ± 1.4 vs. 8.6 ± 2.3, p = 0.011) and HIV risk reduction knowledge (11.0 ± 2.2 vs. 9.8 ± 3.1, p = 0.008) at baseline. However, at the time of first follow-up, the follow-up group performed better only in the basic HIV information domain (10.8 ± 0.5 vs. 10.5 ± 1.0, p = 0.023). Moreover, self-reported condom use and injection risk behaviors at baseline were not found different (p > 0.05) between the retained and lost to follow-up individuals in the intervention group.

### Characteristics of group early follow-up and late follow-up at the second follow-up

The median and inter-quartile range of second follow-up time was 10.7 and 10.9 months in the intervention group, 5.9 and 5.7 months in the control group. In order to determine if there was a difference in those who followed up earlier versus later at the second follow-up, these two groups were compared. Participants that had earlier follow-up were not significantly different from individuals who were followed up later in terms of age, sex, marital status, ethnicity, education, occupation, home province, and income. There were no significant differences between the early and late follow-up groups in the three HIV knowledge domains at the second follow-up. No difference in condom use or injection risk behaviors was found between the early and late follow-up group at the second follow-up.

## Discussion

Although there have been numerous successes in China's response to the IDU HIV epidemic [13,14], sustainably changing high risk sexual and drug-using behaviors has been difficult. Similar to many parts of the world [38,39], drug users in China represent a marginalized group that is both challenging to identify and whose behavior is difficult to change long-term. Peer-based programs in China have shown promise [17-20], although formal evaluation has been limited. This research project improves on previous peer-based IDU HIV prevention programs through its multiple follow-up points including post-detention community assessment, formalized peer training, and conceptual framework.

The baseline socio-demographic characteristics, HIV knowledge, and sexual and drug using behaviors in this sample are similar to other studies of detained drug users in south China [34,40,41]. The differences in sex, education level, and ethnicity between intervention and control groups are likely due to district-level differences in the composition of the drug availability and drug users. Importantly, there were no significant differences between the two groups in self-reported HIV knowledge or sexual and drug using behaviors at baseline. We found that the peer-based education program improved HIV-related knowledge for the drug users. Participants in the intervention group demonstrated substantial improvements across all domains of HIV knowledge at both follow-up visits, in contrast to minor changes in the control group. This difference was observed despite intervention drug users starting with significantly less education at baseline. Consistent with other studies which have found poor HIV knowledge among detained drug users in China [34,42], this finding demonstrates the limitations of routine behavioral counseling and education in the drug rehabilitation system. Furthermore, multivariate mixed regression analysis revealed that the intervention group did have a sustained impact on HIV knowledge, both in the rehabilitation centers and later in the community. Hence, peer-based interventions may improve drug users' knowledge, while underlying a chance for introducing such interventions for these marginalized and resource-limited populations. Nevertheless, attenuation of intervention effects may occur because of interruptions to the intervention program. Results from this study indicated that the effect on HIV-related knowledge did diminish during the second follow-up. This suggested that additional post-release community services might be needed to help maintain a long-term effect.

Self-reported behaviors assessed at the second follow-up suggest that the peer-based intervention resulted in short-term behavior change. The intervention significantly decreased the frequency of injecting drug use, and showed a trend to increase the frequency of using a new syringe. In terms of sexual behaviors, the frequency of condom use with casual sex partners was significantly higher among the intervention group compared to the control. The lack of significant changes in condom use frequency with sex workers or steady partners could be related to the role play and peer education curricula focusing more on casual sex partners. Although there is no precedent for effective peer-based IDU HIV prevention in China, studies from outside of China have also been successful at increasing HIV knowledge and promoting behavior changes [23,43,44]. However, a novel aspect is concern that intervention effects might be largely explained by how they affect the peer educators themselves [45]. Further research for peer-based educations needs to elucidate the mechanism of peer educator influence in order to scale up these interventions.

Ensuring appropriate follow-up of IDUs is critical for effective HIV prevention programs, and this research project had a large number of individuals lost to follow-up. However, compared to other literature on detained drug users in China, our follow-up rates were relatively high [17]. The higher loss to follow-up among women, non-local residents, and those with less HIV knowledge has implications for interpreting the effectiveness of the intervention. The tendency for those who had follow-up to report more HIV knowledge at baseline compared to those without follow-up suggests that the intervention may have only had an impact among a small number of drug users.

There are several limitations to this study worthy of further discussion. The low follow-up rates and differences between those who did and did not receive follow-up limit the generalizability of the results. Although the study was designed as a pilot, there were both measured and unmeasured differences between the control and intervention groups that could introduce bias. For example, differences in baseline routine HIV/STI education programs at each of the sites could affect changes in HIV knowledge and behaviors over time. In addition, no biomarker data were collected among participants.

Since HIV and STI prevalence are higher in many prison settings [46,47], detention settings have been increasingly used as a location for HIV/STI interventions. This study demonstrates the feasibility of peer-based drug user HIV interventions in Chinese detention settings, with several important caveats. This study shows how detention and public health authorities can successfully collaborate to create effective HIV prevention programs for IDUs in China, laying the foundation for further research and action. Studies that incorporate biomarkers and randomization would be useful for extending the preliminary findings reported here. While the Chinese drug rehabilitation infrastructure has been criticized by international human rights groups [48], the administrative systems of detention are likely to persist for some time [30].

## Conclusions

This study shows that rehabilitation centers may be a useful location for providing behavioral HIV/STI prevention services and referral of individuals to community-based programs upon release. More research is needed on behalf of detained drug users in China who have complex social, medical, and legal needs.

## Competing interests

The authors declare that they have no competing interests.

## Authors' contributions

SYS contributed to the study's organization and operation, conducted investigation, intervention, follow-up survey, data compilation, statistical analyses, drafted the manuscript. ZBZ contributed to the study and questionnaire design, conducted coordination. AHL conceptualized and designed the study and questionnaire, obtained funding, conducted organization and coordination. JDT and HC conducted statistical analyses, wrote and revised the manuscript. GRZ conducted a portion of statistical analyses and mainly revised the manuscript.

All authors provided critical revision of the manuscript for important intellectual content, read and approved the final manuscript.

## Pre-publication history

The pre-publication history for this paper can be accessed here:

http://www.biomedcentral.com/1471-2458/11/693/prepub
